# *In situ* simulation in pediatrics

**DOI:** 10.1186/1824-7288-40-S1-A10

**Published:** 2014-08-11

**Authors:** Marco de Luca

**Affiliations:** 1 Emergency Department, Meyer Children’s Hospital, Florence, 50139, Italy

## 

The increasing speed of medicine updating required a change in the training system: the classic teaching mode "see one-do one" is increasingly called into question for ethical reasons in order to protect patients from possible damages deriving from health personnel education [1]. This concept finds its implication in pediatric patients, where any unnecessary suffering must be avoided: for this reason new methodologies based on simulation have been introduced to prevent this type of problem.

Simulation is a technique that allows replacing or amplifying real experiences with guided experiences, artificially designed, which evoke or replicate substantial aspects of the real world in an entirely interactive situation. The growing interest in this approach is due to three main factors: the development of simulator technologies, the evolution of medical education understood as a set of competences in the field of knowledge, skills and behavior and finally the increasing attention to limit errors in team work.

Simulation in medicine is not only a training tool, but also a way of reducing clinical risk and of improving patient safety.

Pediatric emergencies are an example of how simulation can actually be a tool for health workers to achieve a performance improvement in medical assistance [2]. Pediatric emergency events are clinically rare, high-risk, and frequently involve the simultaneous presence of several subjects: pediatricians, nurses, specialists and the patient’s family.

*In situ* simulation is a particular form of simulation, distinct from simulation conducted in a simulation center: it may be defined as, “Simulations that occur in the actual clinical environment and whose participants are on-duty clinical providers during their actual workday.” The required materials can either be used specifically for simulation and taken each time to the site of the scenario, or they can be routinely available. *In situ* simulation training offers many advantages: it gives the opportunity to test systems, to reinforce training in authentic ways, and also to identify hazards and deficiencies in the clinical systems, in the environment and in the provider team [3].
